# Core-shell NaGdF_4_@CaCO_3_ nanoparticles for enhanced magnetic resonance/ultrasonic dual-modal imaging via tumor acidic micro-enviroment triggering

**DOI:** 10.1038/s41598-017-05395-w

**Published:** 2017-07-14

**Authors:** Zuwu Wei, Xiao Lin, Ming Wu, Bixing Zhao, Ruhui Lin, Da Zhang, Yun Zhang, Gang Liu, Xiaolong Liu, Jingfeng Liu

**Affiliations:** 1 The United Innovation of Mengchao Hepatobiliary Technology Key Laboratory of Fujian Province, Mengchao Hepatobiliary Hospital of Fujian Medical University, Fuzhou, 350025 People’s Republic of China; 2 The Liver Center of Fujian Province, Fujian Medical University, Fuzhou, 350025 People’s Republic of China; 3 Liver Disease Center, the First Affiliated Hospital of Fujian Medical University, Fuzhou, 350025 People’s Republic of China; 4 Academy of Integrative Medicine, Fujian University of Traditional Chinese Medicine, Fuzhou, 350122 People’s Republic of China; 5 Key Laboratory of Design and Assembly of Functional Nanostructures, Fujian Institute of Research on the Structure of Matter, Chinese Academy of Sciences, Fuzhou, 350002 People’s Republic of China; 6 Center for Molecular Imaging and Translational Medicine, Xiamen University, Xiamen, 361102 People’s Republic of China

## Abstract

For cancer diagnosis, a paramount challenge still exists in the exploring of methods that can precisely discriminate tumor tissues from their surrounding healthy tissues with a high target-to-background signal ratio. Here, we report a NaGdF_4_@CaCO_3_-PEG core-shell nanoparticle which has the tumor acidic microenvironment enhanced imaging signals of ultrasound and magnetic resonance. Under the acidic conditions, the CaCO_3_ shell will gradually dissolve which then facilitate the interaction of NaGdF_4_ with the external aqueous environment to enhance water proton relaxation. Meanwhile, the CO_2_ bubbles generated by the CaCO_3_ dissolvement will generate strong elastic echo for US detection. The core-shell structure of NaGdF_4_@CaCO_3_-PEG can be observed by TEM, and its composition can be determined by STEM. The acid triggered generation of CO_2_ bubbles and the enhancement of MRI signal could be demonstrated *in vitro*, and the excellent dual-modal magnetic resonance/ultrasonic cancer imaging abilities of NaGdF_4_@CaCO_3_-PEG could be also proved at the tumor site *in vivo*. The here described proof-of-concept nanoparticles with pH triggered magnetic resonance/ultrasonic dual-modal imaging enhancement, may serve as a useful guide to develop various molecular imaging strategies for cancer diagnosis in the future.

## Introduction

Cancer is becoming to one of the most dreaded disease and still remains as a major threat to human life^1^. The rising burden is ascribed to population growth, aging and an adoption of cancer-associated lifestyle choices including smoking, physical inactivity, etc^1, 2^. Even worse, most patients were diagnosed at a later or advanced stage, which results in poor prognosis^1, 3^. Therefore, early diagnosis of cancer is crucial for timely therapy to prevent the potential risk of cancer metastasis and improve the long-term survival. Many non-invasive biomedical imaging techniques have been applied in the diagnosis of cancer including magnetic resonance imaging (MRI)^4–9^, ultrasound imaging (US)^10, 11^, positron emission tomography (PET)^12–15^, and computed X-ray tomography (CT)^16–19^, etc. Among the clinically applied diagnostic modalities, MRI has a high potential to image the tissue pathological changes, as it could safely provide high spatial resolution information^20^. On the other hand, as a noninvasive real-time imaging modality, US has serveal advantages such as high safety, low cost, and easy accessed by the public. However, the sensitivity of conventional MRI and US strategies heavily rely on contrast agents (CAs), such as the widely used paramagnetic gadolinium ions (Gd-(DTPA)]^−2^ (Magnevist) and [Gd-(DOTA)]^−1^ (Dotarem)^21–23^, or gas-filled echogenic microbubbles^24, 25^, that are always “on”, emitting constant signals regardless of their proximity or interaction with target tissues, cells, or environmental markers. As a result, a large volume of nonspecific signal which might lead to a poor signal-to-noise ratio, makes the anatomical features of interested tissue difficult to distinguish. A more attractive contrast agent whose signal should be switched from OFF to ON in response to specific biological stimulus, which will further maximize the signals of targets and minimize the background signals, which in turn could improve the sensitivity and specificity^26–29^.

Several activatable CAs that respond to tumour-related factors, such as pH and redox potential, have been developed for tumour-specific MRI or US. For example, Mi *et al*. have reported a MRI contrast agent that rapidly amplify the magnetic resonance signals in response to pH via releasing confined Mn^2+^ ions from pH-sensitive calcium phosphate (CaP) nanoparticles to the aqueous environment^30^. Min *et al*. developed the calcium carbonate (CaCO_3_) nanopartciles which exhibited strong echogenic signals at tumoral acid pH by producing CO_2_ bubbles and showed excellent echo persistence^10^. Even so, some intrinsic drawbacks of US and MRI still cannot be avoided, for example, US has a poor tissue discrimination ability while MRI cannot provide real-time images and usually time consuming. Thus, developing a sensitive dual modal imaging (US and MRI) agent would not only make them as favorable tools for precisely visualizing biological and physiological changes with high signal-to-noise ratio, but also would render synergistic efficacy to overcome their own inherent limitations^31^.

As a MRI contrast agent, various size of NaGdF_4_ nanoparticles (NPs) with well-defined size distributions could be readily synthesized via pyrolysis methods^32–35^. Their MRI performances have been demonstrated to increase with the decreasing of the NPs’ size, attributed to the increased number of surface Gd^3+^ ions relative to the core ions. Therefore, it will be very useful to synthesize ultrasmall NaGdF_4_ NPs, e.g., smaller than 5 nm, to provide better MRI signals^6^. Furthermore, compared with the Gd-(DTPA) or Gd-(DOTA), the renal clearable Gd-based NPs would have better biosafty since it could not induce the excessive Gd^3+^ ion leakage to cause biological toxicity^36, 37^, therefore it might be a more promising candidate for disease diagnosis. As for a new type of US contrast agent, CaCO_3_ nanoparticles with rigid structure can penetrate into host tumoral environments for cancer imaging, while the frequently used gas-filled microbubbles suffer from inherent drawbacks, such as low stability, short half-life in blood and low penetration ability due to the large size, which is limited to the imaging of intravascular structures.

**Figure 1:**
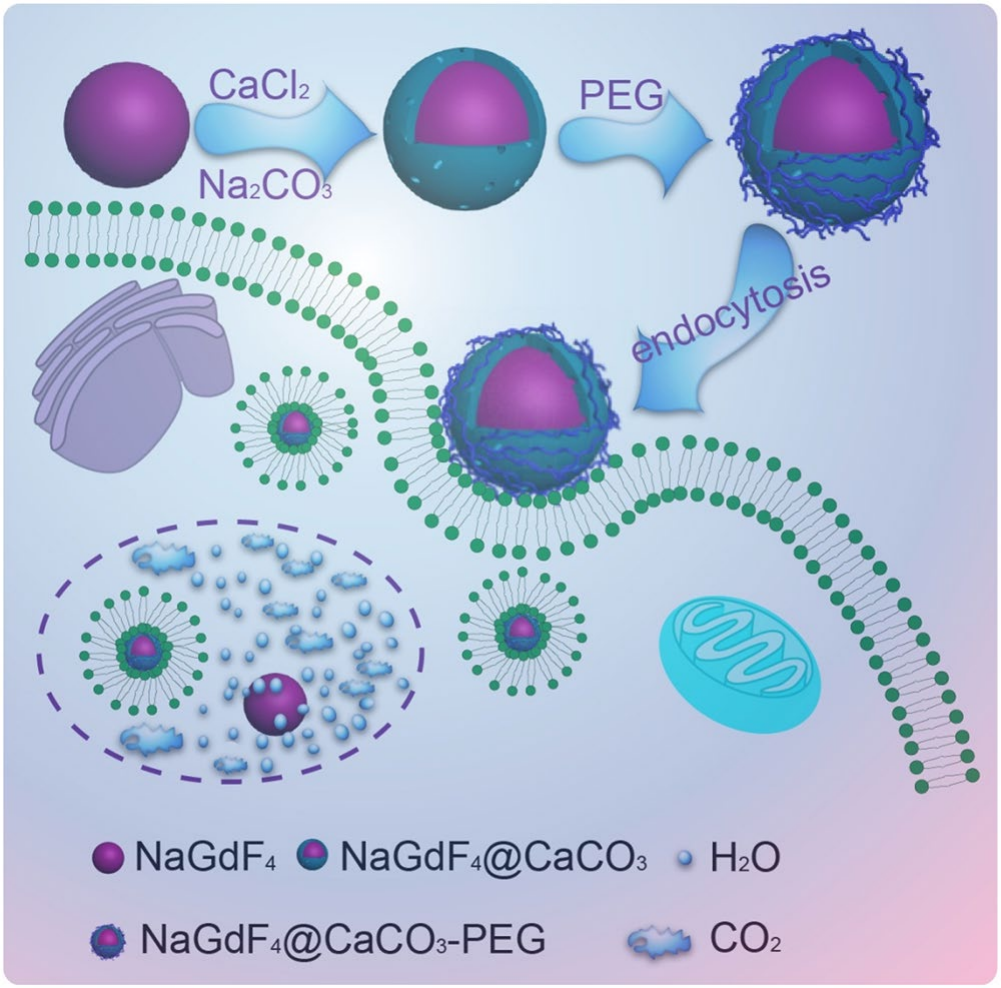
Schematic illustration of the synthesis of NaGdF_4_@CaCO_3_-PEG nanoparticle and its bioimaging application.

Herein, we designed a pH-responsive nanoparticle which could significantly enhance the contrast of MRI and US signals in tumor as illustrated in Fig. 1. The shell of CaCO_3_ was deposited onto the core surface of NaGdF_4_ nanoparticles through the microemulsion method; in addition, physicochemical properties of nanoscale systems, such as size, dispersibility, and toxicity were systematically analyzed; furthermore, the US and MRI imaging enhanced efficiency were both evaluated *in vitro* and *in vivo*, which clearly proved that our probe could be utilized for sensitive and specific tumor imaging with responding to extracellular acidic microenvironments.

## Results and Discussion

### Characterization of NaGdF_4_@CaCO_3_-PEG

**Figure 2:**
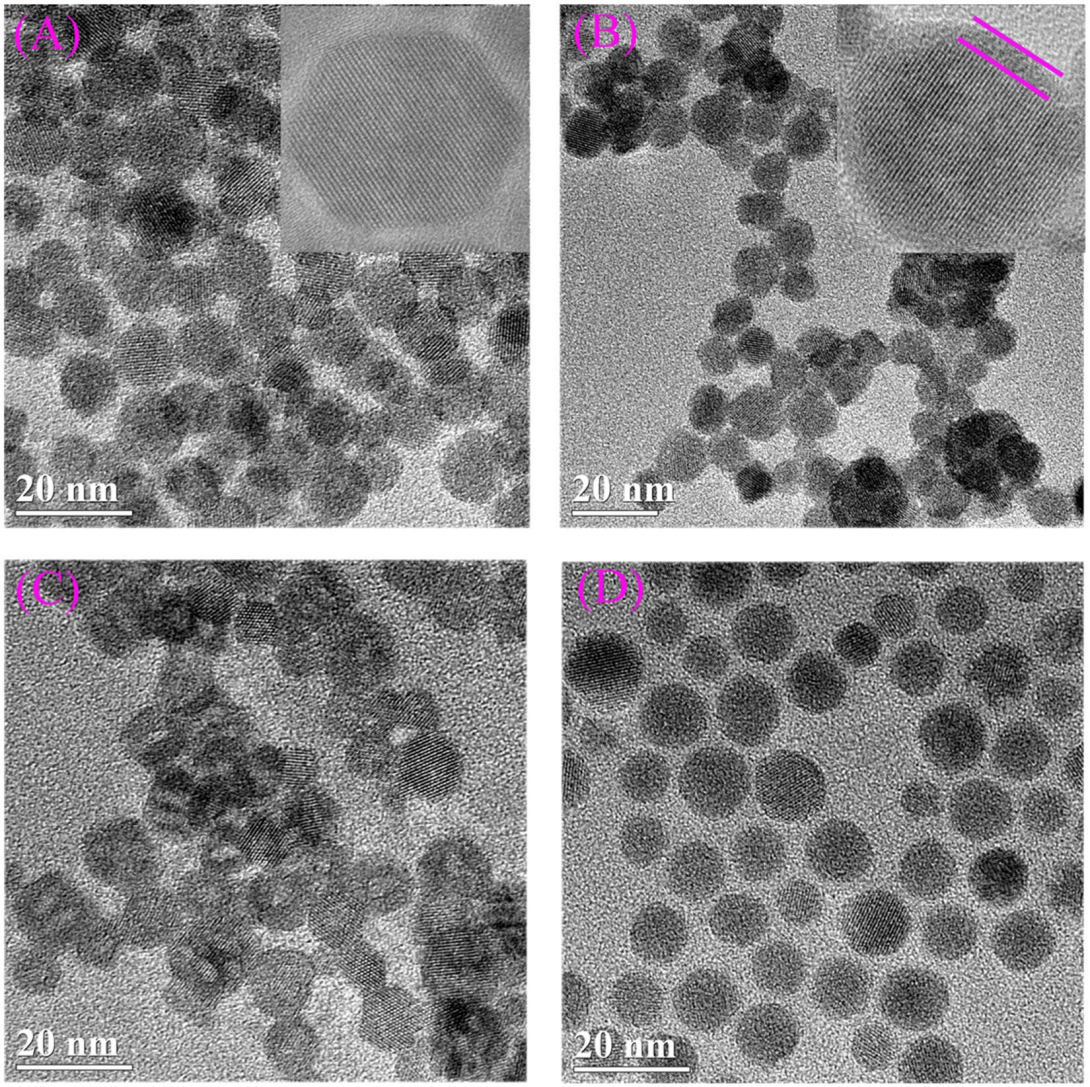
Structural characterization of nanoparticles. NaGdF_4_ dispersed in cyclohexane (**A**); NaGdF_4_@CaCO_3_ (**B**) and NaGdF_4_@CaCO_3_-PEG (**C**) dispersed in H_2_O; and NaGdF_4_@CaCO_3_-PEG dispersed in PBS (pH 5.0).

As shown in Fig. 1, monodispersed spherical NaGdF_4_ nanocrystals were chosen as the morphology-deciding template to obtain magnetic nanoparticles, which were fabricated by thermal decomposition method using rare earth perchlorate as the RE precursor. From TEM images (Fig. 2A), the synthesized NaGdF_4_ exhibit a very well particle size distribution around 8~10 nm, with a regular spherical morphology. A thin layer of dense CaCO_3_ was deposited onto the surface of NaGdF_4_ to form NaGdF_4_@CaCO_3_ core/shell nanoparticles by the well-known microemulsion method, which could further transfer the hydrophobic NaGdF_4_ nanoparticle into aqueous soltion. In detail, the CaCl_2_ aqueous solution was first added into the cyclohexane solution of hydrophobic NaGdF_4_, while triton X-100, 1-hexanol were used as nonionic surfactants to create a stable water-in-oil emulsion after vigorous stirring. At this stage, the hydrophobic NaGdF_4_ will transfer from oil phase to water droplets as the surfactants can self-assemble on these nanoparticles. Meanwhile, the hydrophilic groups (hydroxyl) of the surfactants has a well affinity with Ca^2+^ which could recruit these ions on the surfaces of the NaGdF_4_ nanoparticles. After adding Na_2_CO_3_ aqueous solution, the precipitation reaction between Ca^2+^ and CO_3_^2−^ therefore produces a layer of CaCO_3_ on the NaGdF_4_ nanoparticles. The obtained nanoparticles show a size of 10~12 nm, as well as with aspherical-like morphology. Then, PEG_8000_ was adsorbed on the surface of NaGdF_4_@CaCO_3_ nanoparticles through Van der Waals’ force, which could award good water dispersibility. Element mapping of NaGdF_4_@CaCO_3_-PEG with a thin or thick shell all shows the co-existence of Gd, F and Ca elements (Figure S1), which exhibit a much higher Ca signal compared to the core only NaGdF_4_ nanoparticles. However, the Ca signal on the surface of NaGdF_4_ is very weak on the outlayer of NaGdF_4_ core due to the very small size of NaGdF_4_ core (10 nm) and the very thin CaCO_3_ coating layer (2 nm). Nevertheless, we still ensure that the CaCO_3_ is on the shell because this layer is amorphous, while the NaGdF_4_ core is highly crystal with noticeable lattice, as shown in the enlarged image inserted in Fig. 2B. In addition, the average size of the NaGdF_4_ in cyclohexane was further determined to be 8 ± 2 nm by the DLS experiments (Figure S2), which is consistent with the TEM results. After depositon of CaCO_3_, the average hydrodynamic size of the NaGdF_4_@CaCO_3_ and NaGdF_4_@CaCO_3_-PEG in water were increased to 100 ± 10 and 120 ± 15 nm, respectively, which may be due to the formation of a hydration layer on the surface of nanoparticles or the formation of small aggregations.

**Figure 3:**
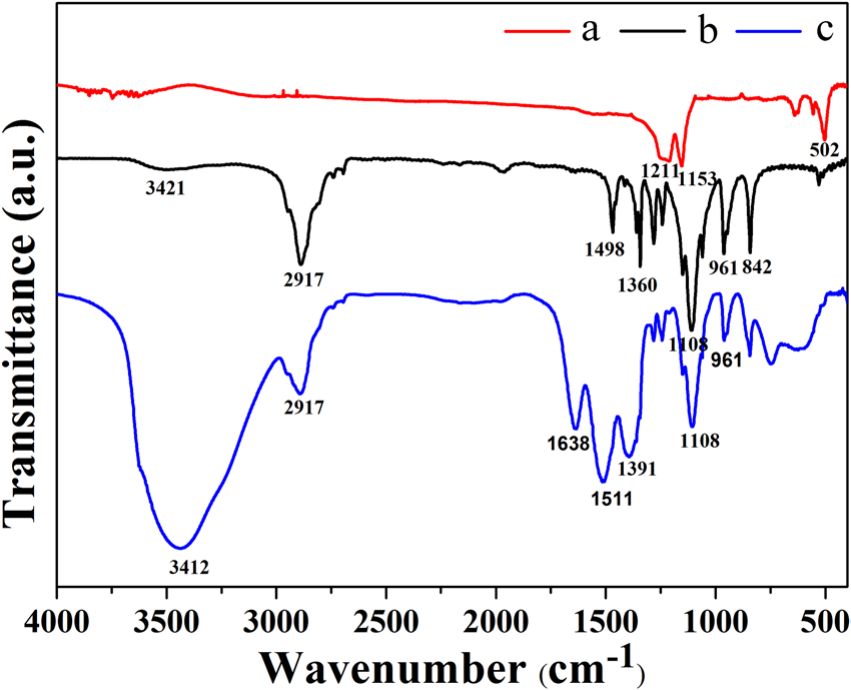
FT-IR spectra of NaGdF_4_@CaCO_3_ (a), PEG_8000_ (b) and NaGdF_4_@CaCO_3_-PEG (c).

Furthermore, the successful surface PEGlyation of NaGdF_4_@CaCO_3_ was also confirmed by FT-IR spectroscopy (Fig. 3). For analyzing the spectrum of PEG_8000_, the peaks at around 2917 cm^−1^ and 1108 cm^−1^ were observed corresponding to the CH_2_ stretching and vibration groups. These two peaks could be also observed in the spectra of NaGdF_4_@CaCO_3_-PEG, in addition to the typical peaks of NaGdF_4_@CaCO_3_ at 502 cm^−1^. These results suggest that the PEG_8000_ was successfully modified on the surface of NaGdF_4_@CaCO_3_. To make the CaCO_3_ coating more evident form the IR spectra, we re-fabricated another NaGdF_4_@CaCO_3_ with a thicker shell *via* increasing the feeding amount of CaCl_2_, and analysis their surface groups through FT-IR measurement. The characteristic IR absorption peaks of CaCO_3_ at 1430, 876, 712 cm^−1^ can be clearly presented in thick layered NaGdF_4_@CaCO_3_ (Figure S3).

Before further bio-imaging applications, it is necessary to determine the cytotoxicity of these nanoparticles. To assess the *in vitro* cytotoxicity of NaGdF_4_@CaCO_3_-PEG, the standard Cell Counting Kit (CCK-8) assay and live/dead staining were conducted by using LN3 and NIH3T3 cell lines. As shown in Figure S4, cell viability is not affected by the NaGdF_4_@CaCO_3_-PEG in the concentration ranged from 0 to 400 μg∙mL^−1^, which suggested that the NaGdF_4_@CaCO_3_-PEG has low cytotoxicity so that it could be further applied for bio-imaging.

### ***In Vitro*** Ultrasound Contrast Enhancement Ability of NaGdF_4_@CaCO_3_-PEG

**Figure 4:**
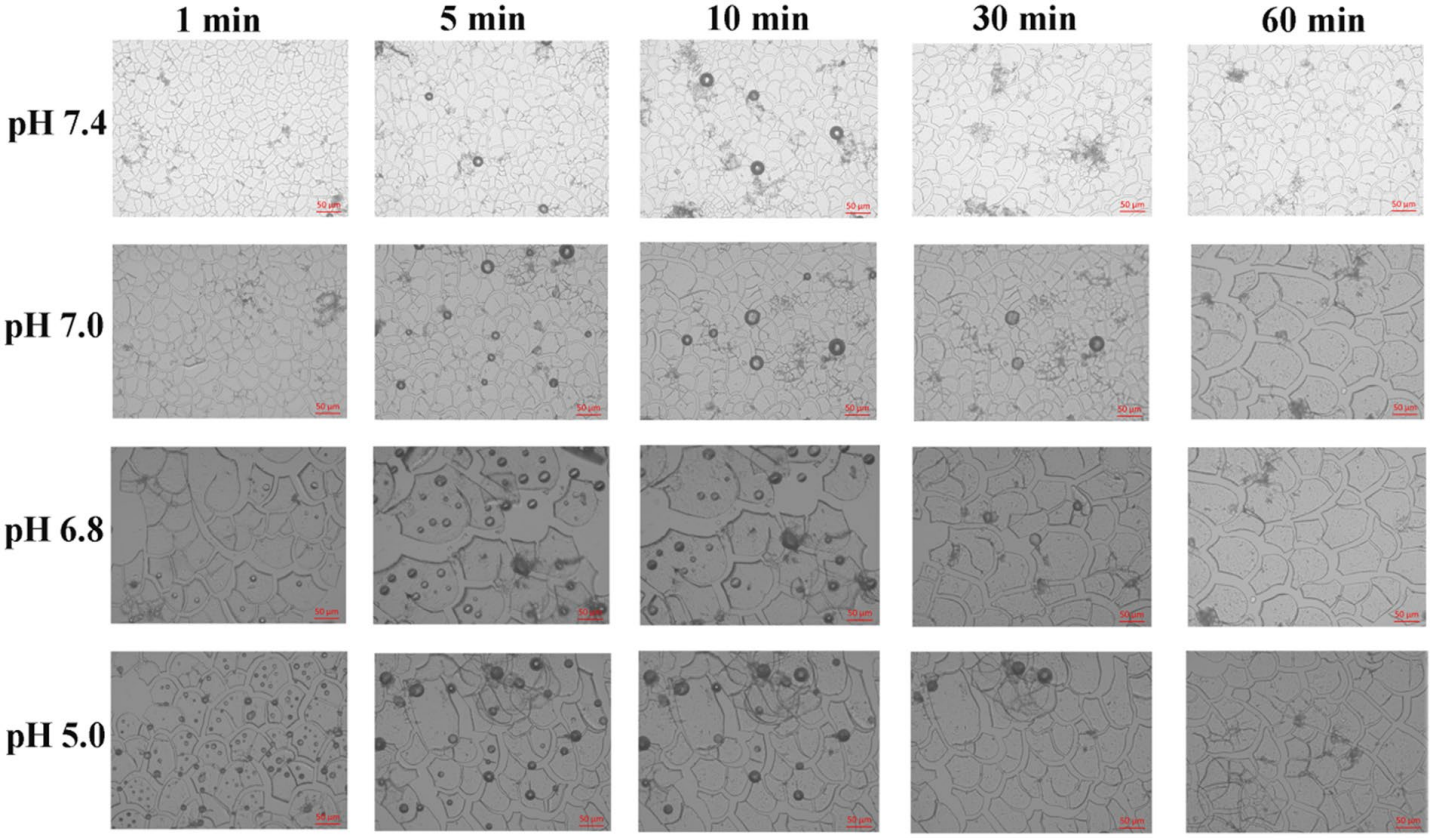
Optical micrographs of CO_2_-generation profiles of NaGdF_4_@CaCO_3_-PEG incubated in PBS at different pH conditions (pH 5.0, pH 6.8, pH 7.0 and pH 7.4) for 60 min.

First, the characteristics of CO_2_ gas generation by NaGdF_4_@CaCO_3_-PEG was studied through detecting the bubble generation from the particles in PBS buffer with different pH values (pH 7.4, 7.0, 6.8 and 5.0). As shown in Fig. 4, initially, NaGdF_4_@CaCO_3_-PEG nanoparticles generate few bubbles at pH 7.4, 7.0 and 6.8, but significantly more bubble generation could be observed at pH 5.0. The degree of bubble generation continuously decreased at pH 5.0 over time, but the bubble generation was first increased and then decreased at pH 7.4, 7.0 and 6.8. The number of bubbles in pH 5.0 was counted to *ca*. 119, which was much higher than that in pH 7.4 (almost no bubbles), after 1 min incubation. However, with increasing the time, the number of bubbles was dramatically decreased at pH 5.0, and only 2 bubbles could be observed after 60 mins incubation (Figure S5). It is noteworthy that bubble generation at lower pH was higher than that at slightly stronger pH at all-time points. This result suggested that the NaGdF_4_@CaCO_3_-PEG might enhance US signal in the low pH environment by gas generation, since CaCO_3_ could generate more CO_2_ gas in an acidic environment by improved dissolution, which would be beneficial for imaging the tumor due to a little acidic microenvironment of the tumor micro-environment.

**Figure 5:**
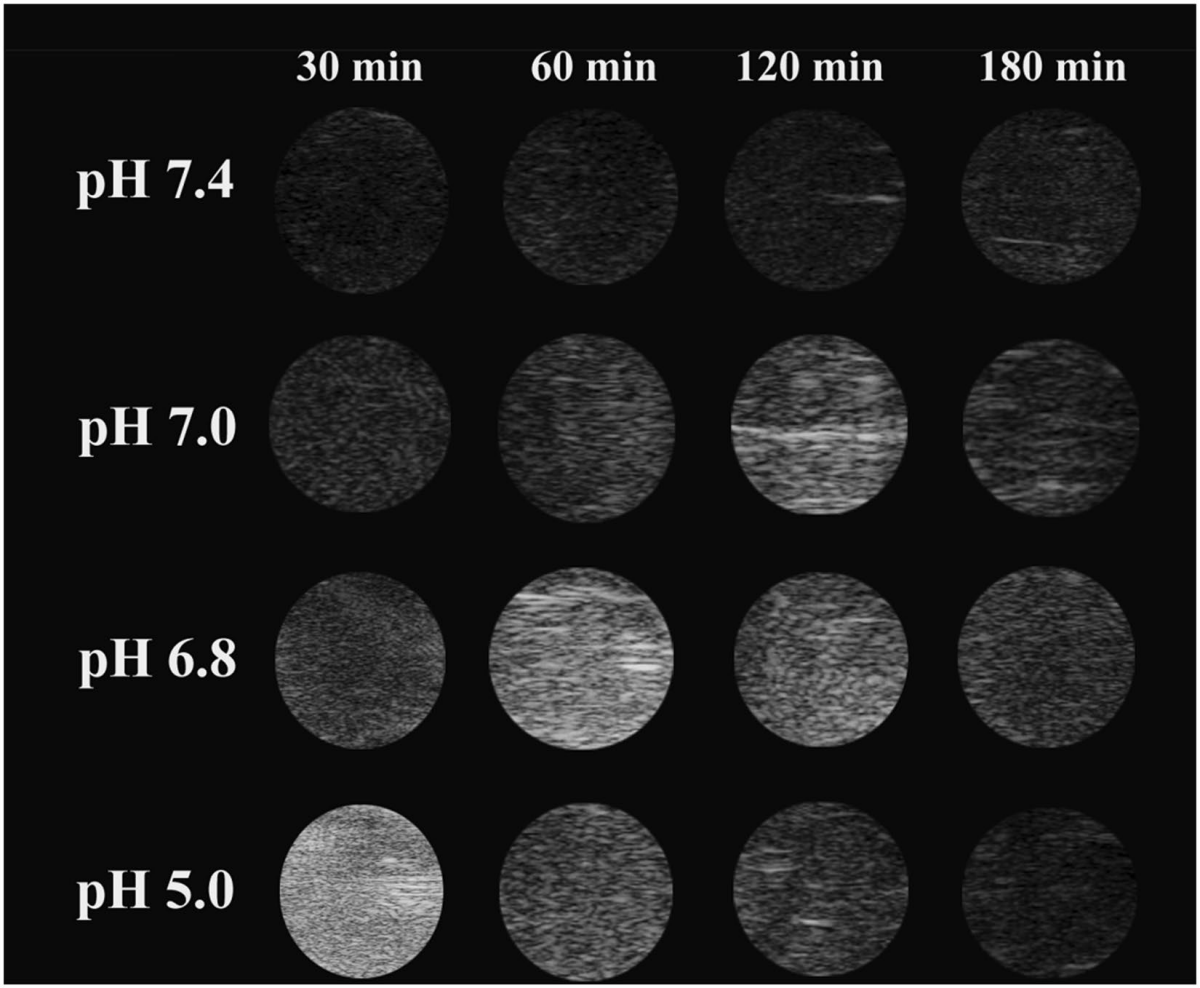
*In vitro* US images from NaGdF_4_@CaCO_3_-PEG at various pH (pH 5.0, pH 6.8, pH 7.0 and pH 7.4) conditions along with time.

Next, we measured the *in vitro* echogenic performance of the NaGdF_4_@CaCO_3_-PEG as a function of pH using an aqueous dispersion test at 37 °C. As shown in Fig. 5, the NaGdF_4_@CaCO_3_-PEG at pH 7.4 showed no significant contrast signals under a US field, most likely because the NaGdF_4_@CaCO_3_-PEG did not release sufficient CO_2_ bubbles for echogenic reflection. This result is well consistent with the results of the pH-dependent CO_2_ generation (Fig. 4). In contrast, US contrast images from the NaGdF_4_@CaCO_3_-PEG showed first increased and then decreased signal at all-time points at weakly acidic pH conditions (pH 7.0 and 6.8). This is a well understood phenomenon in which the enhanced echo intensity at weakly pH levels is ascribed to the relaxedly facilitated formation of CO_2_ bubbles. Due to faster dissolution of CaCO_3_ phases at pH 5.0, the echo improved immediately and faded away earlier than at higher pH. The excellent echo persistence at weak acidic pH can be ascribed to the relaxedly ionization of CaCO_3_ solid phases. The contrast enhancement that derives from the NaGdF_4_@CaCO_3_-PEG at lower pH suggested that generation of CO_2_ bubbles was responsible for echogenic US resonation. US imaging results were further quantitatively analyzed using region-of-interest (ROI) gray value quantification (Figure S6A). The gray value in pH 5.0 and pH 7.4 was respectively determined to be 139.2 ± 10.7 and 14.2 ± 1.2, after 30 min incubation. This result is in accordance with the CO_2_ bubbles generation. Meanwhile, in order to further confirm that the US signal was derived from the dissolution of CaCO_3_ at acidic solution rather than the NaGdF_4_ itself, we tested US signal of the core-only (NaGdF_4_) nanoparticle at pH 5.0 and 7.4, and the results are shown in Figure S7. NaGdF_4_ nanoparticle showed no US signal at both pH 5.0 and 7.4, due to no CO_2_ gas generation from NaGdF_4_ nanoparticle. The feature of gas-generating at a broad range of weak acidic pH would be propitious to US imaging of tumors, which have tissue heterogeneity and diverse acidic pH levels.

### pH-Dependent *In Vitro* MRI Properties of NaGdF_4_@CaCO3-PEG

**Figure 6:**
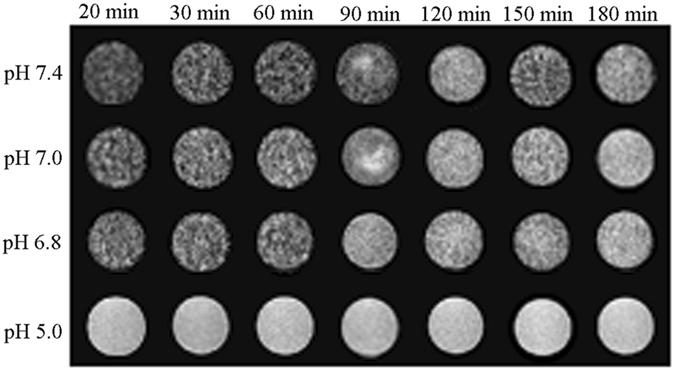
*In vitro* MR images from NaGdF_4_@CaCO_3_-PEG at various pH (5.0, 6.8, 7.0 and 7.4) conditions along with the time.

Since the doping of Gd^3+^-ions, NaGdF_4_@CaCO_3_-PEG could act as a T_1_ MRI contrast agent as well. T_1_-weighted MR images of the NaGdF_4_@CaCO_3_-PEG showed enhancing signal intensity when Gd^3+^ concentrations were increased. Firstly, the T_1_-weighted MR image of the NaGdF_4_@CaCO_3_-PEG was studied at different pH conditions. As shown in Fig. 6, the NaGdF_4_@CaCO_3_-PEG at pH 7.4 showed no significant enhanced contrast signals under a MRI field, most likely because the shell of CaCO_3_ did not decompose so that water molecular could not access to NaGdF_4_ core. In contrast, MRI contrast images from the NaGdF_4_@CaCO_3_-PEG were slightly enhanced at weakly acidic pH conditions (pH 7.0, 6.8). It is noteworthy that we demonstrated strongest MRI contrast images at pH 5.0, which might due to the decomposition of the CaCO_3_ shell so that water molecular could access to the NaGdF_4_ core. To further demonstrate this mechanism, we compared the T_1_-MRI of NaGdF_4_@CaCO_3_-PEG to that of core-only (NaGdF_4_) nanoparticles at pH 7.4, and the results are shown in Figure S6. Of course, NaGdF_4_ showed a much brighter MR image than NaGdF_4_@CaCO_3_-PEG at pH 7.4. With the increasing of incubation time, the contrast of MRI images gradually enhanced at pH 7.2 and 6.8, but almost no change at pH 7.4. MRI imaging results were then quantitatively analyzed using region-of-interest (ROI) gray value quantification (Figure S6B). The gray value in pH 5.0 and pH 7.4 was respectively determined to be 182.2 ± 10.1 and 79.9 ± 3.1, after 20 min incubation. These data are very consistent with the theory that the contrast of T_1_ MRI imaging is closely related with water accessibility, and the enhancement of contrast associated with sufficient water contacting.

**Figure 7:**
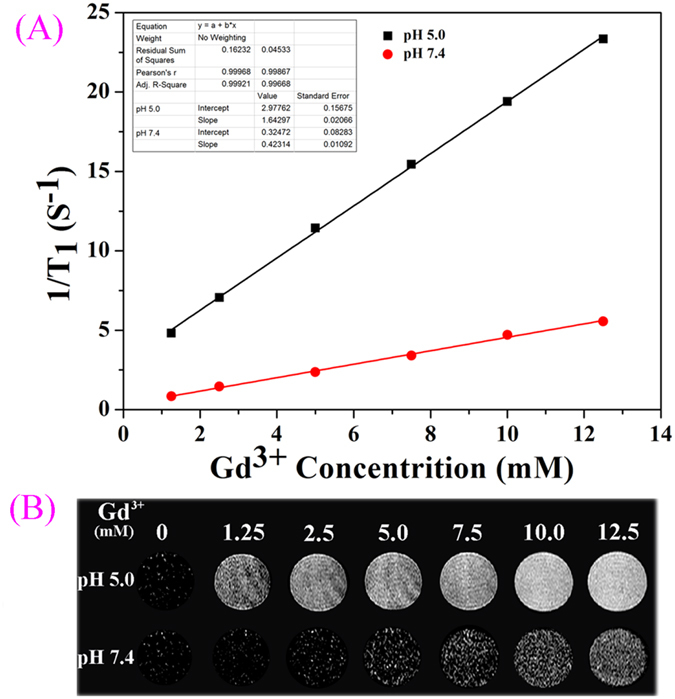
T_1_-Weighted MR images of various Gd^3+^ concentrations of NaGdF_4_@CaCO_3_-PEG (**A**); relaxation rate r_1_ (1/T_1_) against different Gd^3+^ concentrations of NaGdF_4_@CaCO_3_-PEG (**B**).

In addition, the T_1_ value of the NaGdF_4_@CaCO_3_-PEG was evaluated by using a 0.5 T MRI scanner to determine whether the pH can influence the T_1_-weighted MR imaging performance (Fig. 7). As shown in Fig. 7A, the longitudinal (T_1_) were measured at 0.5 T magnetic field based on the Gd concentration of NaGdF_4_@CaCO_3_-PEG at pH 7.4 and 5.0, and the relaxivities were determined to be 0.42 mM^−1^ ∙s^−1^ and 1.64 mM^−1^ ·s^−1^, respectively. At pH 5.0, it showed about 4 folds higher relaxivity than that at pH 7.4, which further proofed that the T_1_-weighted MR imaging enhancement of the NaGdF_4_@CaCO_3_-PEG was influenced by pH levels. At the same time, Fig. 7B shows the magnetic resonance signal enhancing capability of the NaGdF_4_@CaCO_3_-PEG nanoparticles as a function of Gd concentration ranging from 1.25 to 12.5 mM. Compared with water (Gd, 0 mM), the measured T_1_-weighted image contrast gradually increased with the increasing of Gd concentrations.

### *In Vivo* US Imaging of Tumor with NaGdF_4_@CaCO3-PEG

**Figure 8:**
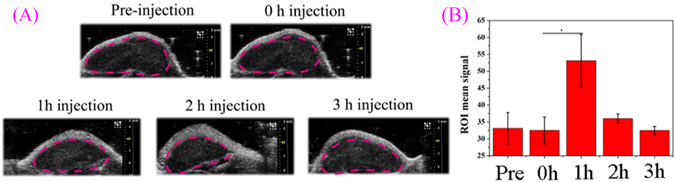
*In vivo* US imaging of the LN3 tumor (red dashed circles) by intratumoral injection of NaGdF_4_@CaCO_3_-PEG (**A**); the gray values of mice tumor (region of interest as indicated in **A**), p values were calculated using GraphPad Prism 6 (*p < 0.05, **p < 0.01, ***p < 0.001; n = 3).

To verify the potential of NaGdF_4_@CaCO_3_-PEG for US imaging of tumor, we executed an intra-tumor injection of NaGdF_4_@CaCO_3_-PEG dispersion into LN3 tumor-xenograft-bearing nude mice and monitored the US images as a function of time (Fig. 8). After injection right away, we couldn’t acquire any contrast enhancment of the US signal. It is gratifying that enhancement of US signals was obtained after 1 h of injection, and this contrast enhancement maintained more than 2 h. In addition, this phenomenon is very consistent with the *in vitro* experiments. US imaging results were then quantitatively analyzed using region-of-interest (ROI) gray value quantification. As shown in Fig. 8B, the gray value of tumor site was increased from 32.5 ± 3.9 (0 h post-injection) to 53.1 ± 7.8 (1 h post-injection), and still maintained at 38.7 ± 1.35 after 2 h injection. Therefore, we can conclude that the NaGdF_4_@CaCO_3_-PEG could generate bubbles in tumor tissues then produce sufficient echogenic reflectivity under a US field.

### *In Vivo* MR Imaging of Tumor with using NaGdF_4_@CaCO_3_-PEG

**Figure 9:**
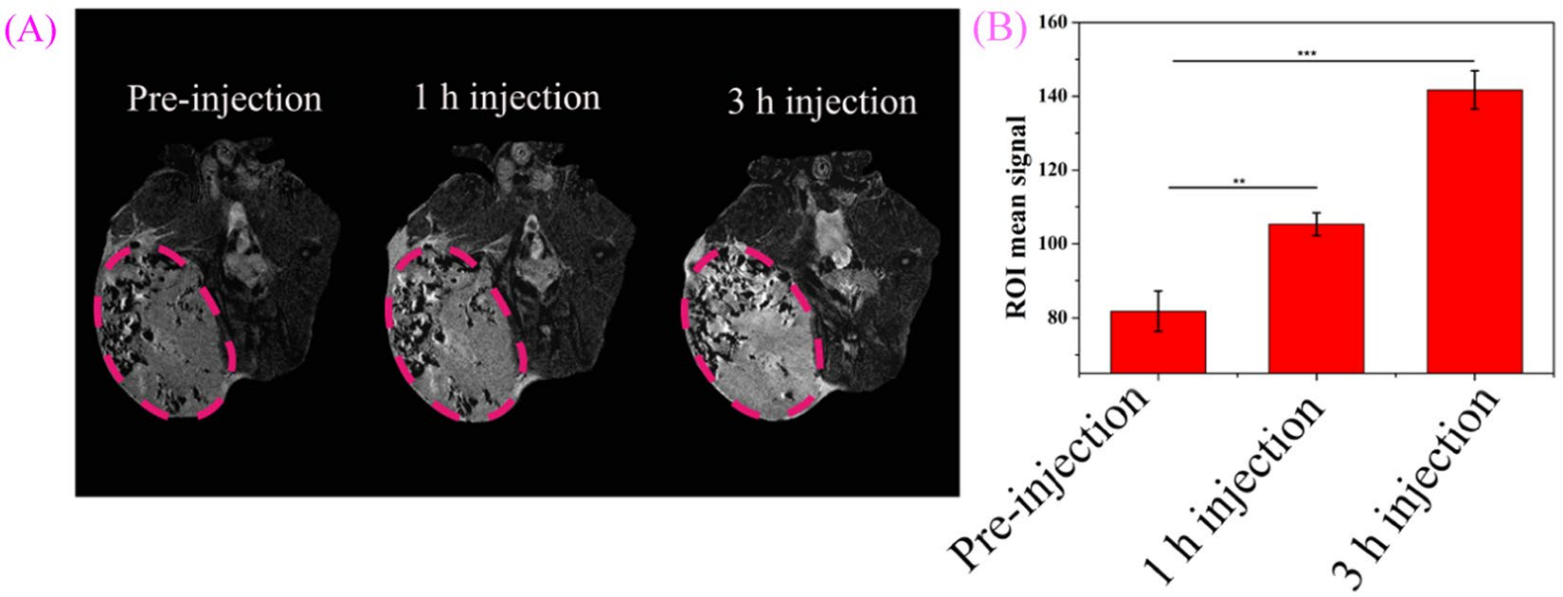
*In vivo* MR imaging of the LN3 tumor (red dashed circles) by intratumoral injection of NaGdF_4_@CaCO_3_-PEG (**A**); the gray values of mice tumor (region of interest as indicated in **A**), p values were calculated using GraphPad Prism 6 (*p < 0.05, **p < 0.01, ***p < 0.001; n = 3).

NaGdF_4_@CaCO_3_-PEG nanoparticles which have the advantages of highly efficient of T_1_ contrast enhancement ability may hold great promise to serve as a novel MRI contrast enhancing agent. Therefore, we performed MRI study on tumor-xenograft-bearing nude mice at a 7.0 T clinical scanner by intratumor injection of NaGdF_4_@CaCO_3_-PEG with a dose of 200 μL of 2 mg∙mL^−1^. Right after the injection, the MRI contrast enhancement was not found at tumor site; however, after one hour of intratumor injection, we were delighted to find contrast enhancement of MRI signal and the signal strength remained unchanged over time up to 3 h (Fig. 9A). T_1_ imaging results were then quantitatively analyzed using region-of-interest (ROI) quantification. As shown in Fig. 9B, the gray value of tumor site was increased from 102 (0 h post-injection) to 153 (3 h post-injection). Overall, these results suggested that the NaGdF_4_@CaCO_3_-PEG could act as a potential MRI contrast enhancing agent for tumor imaging.

## Conclusions

In this work, a core-shell nanoparticle of NaGdF_4_@CaCO_3_-PEG was designed as an activatable MR/US dual-modal imaging contrast for cancer diagnosis, which is triggered by the acidic environment. The general idea behind this OFF/ON responsive MR imaging behavior consists of quenching the sphere Gd^3+^ relaxation effects by coating NaGdF_4_ with a layer of hydrophobic CaCO_3_ to limit water availability. At acidic aqueous solution, CaCO_3_ was dissolved to generate CO_2_ bubbles which is used to obtain US signal. Meanwhile, a strong MRI enhancement can be activated upon dissolution of CaCO_3_ and release of the previously silenced NaGdF_4_ into the aqueous solution. *In vivo* results demonstrated the strong dual-modal magnetic resonance/ultrasonic imaging abilities of NaGdF_4_@CaCO_3_-PEG at the tumor site with an acidic environment. We expect that this work may provide a new insight for strategies to design nanomaterials with responsive dual-modal imaging abilities.

## Methods

### Materials

Gd(CH_3_CO_2_)_3_ and PEG_8000_ were purchased from Sigma Aldrich Co., Ltd. Acetone and cyclohexane were obtained from Sinopharm Chemical Reagent Co., Ltd. 1-Octadecene and Methanol was purchased from Alladin Company. Oleic acid was purchased from Alfa Aesar. Cell Counting Kit (CCK-8) was obtained from Dojindo laboratories. Penicillin-streptomycin, fetal bovine serum (FBS), and Dulbecco’s Modified Eagle Medium (DMEM) were purchased from Gibco BRL. All of these materials were used as received without further purification. Ultrapure water was used throughout.

### Synthesis of NaGdF_4_ nanoparticles

The synthesis of NaGdF_4_ nanoparticles is similar to a previous publication about rare-earth up-conversion nanoparticles^34, 38^. In brief, 24 mL of Gd(CH_3_CO_2_)_3_ (0.2 M) was added to a 500 mL of two-necked round-bottomed flask containing 36 mL of oleic acid. When the mixture was heated to 150 °C for 30 min, 84 mL of 1-octadecene was added and then heated to 150 °C to form the Gd-oleate precursor solution. Then, the reaction system was cooled down to 60 °C naturally and a mixture of 8 mL NaOH (1 M) and 16 mL NH_4_F (0.4 M) in methanol was added and stirred for 30 min. The resultant solution was heated at 110 °C for 30 min to remove the methanol, oxygen and water under argon atmosphere. Thereafter, the solution was heated to 300 °C and kept for 1.5 h before cooling down to room temperature. The as-prepared nanoparticles were precipitated by additional of ethanol, collected by centrifugation at 6000 rpm for 3 min, and washed with ethanol for several times. The NaGdF_4_ nanoparticles are re-dispersed in 50 mL of cyclohexane for further application.

### Synthesis of the core-shell NaGdF_4_@CaCO_3_-PEG nanoparticles

The core-shell NaGdF_4_@CaCO_3_ nanoparticles were prepared through a facile microemulsion method with using NaGdF_4_ nanoparticles as templates^39^. Briefly, 5 mL NaGdF_4_ nanoparticles, 10 mL cyclohexane, 3.45 mL Triton X-100, and 3.2 mL 1-hexanol were added to a flask and mixed thoroughly, followed by the addition of a 30 mM solution of calcium chloride (800 μL) to form a well-dispersed water-in-oil emulsion. Then, the solution of sodium carbonate (40 μL, 2.92 M) was added. After keeping moderate stirring for 8 h of the mixture, the as-prepared NaGdF_4_@CaCO_3_ nanoparticles were collected by centrifugation and then re-dispersed in 10 mL of ultrapure water. Thereafter, 2 mL PEG_8000_ (0.2 M) aqueous solution was added and stirred for another 8 h, then the as-prepared nanoparticles were collected by centrifugation at 10000 rpm for 15 min, and re-dispersed in 20 mL of ultrapure water for further application.

### Characterization of the NaGdF_4_@CaCO_3_-PEG nanoparticles

The morphology and the size of the obtained NaGdF_4_@CaCO_3_-PEG nanoparticles were performed on a Tecnai F20 transmission electron microscope (TEM, FEI Company, Hillsboro, OR) with an accelerating voltage of 200 kV^40^. For the TEM experiment, the suspension of nanoparticles was dropped onto a carbon-coated copper grid, followed by drying naturally. Scanning transmission electron microscopy (STEM) was obtained using a JEM-2010 electron microscope (JEOL, Japan) to characterize the chemical composition of NaGdF_4_@CaCO_3_-PEG nanoparticles^40^. Dynamic light scattering (DLS) experiments were recorded at 25 °C on a NanoZS (Malvern Instruments, UK) with a detection angle of 173°, and a 3 mW He–Ne laser operating at a wavelength of 633 nm. FT-IR spectra were performed on a Fourier transform infrared spectrometer (Perkin-Elmer, Spectrum-2000) over the spectral region of 4000 cm^−1^ to 400 cm^−1^ ^41^.

### Statistical analysis

All quantitative data were expressed as the mean ± standard deviation (SD). Graph Pad Prism version 6.0 was used for statistics analysis. Statistical analysis among different groups was performed using student T-Test. The P < 0.05 was considered as statistically significant.

### Animals

All mice (4–5 weeks old; weighing: 18–22 g) were obtained from the Center for Animal Experiment of Fujian Medical University (License No: SCXKmin2012–0002), and housed at constant temperature (22 ± 2 °C) and 60% relative humidity, with a light/dark (hours) cycles of 12/12. All animal procedures were conducted in accordance with the approved guidelines of Animal Ethics Committee of Fujian Medical University.

### Cytotoxicity assay

The LN3 and NIH3T3 were cultured in Dulbecco’s Modified Eagle Medium (DMEM) containing 10% fetal bovine serum and 1% penicillin streptomycin at 37 °C in a humidified atmosphere (5% CO_2_). The *in vitro* cytotoxicity was investigated by the Cell Counting Kit (cck-8) assay. In detail, LN3 and NIH3T3 cells were cultured in a 96-well cell-culture plate at a density of 10^4^ (100 μL) cells per well for 24 or 48 h, respectively. Then, the medium was replaced with 100 μL fresh medium containing various concentrations of NPs. After 24 or 48 h incubation, the medium was removed. Then, 100 μL of fresh medium and 10 μL of CCK-8 were added and incubated for another 2 h. The absorbance was measured by a Bio-Rad Model-680 microplate reader at the wavelength of 450 nm. The cell viability (%) relative to control cells was calculated from following equation: ([Abs]_sample_ − [Abs]_blank_)/([Abs]_control_ − [Abs]_blank_) × 100%, where [Abs]_sample_ and [Abs]_control_ are the absorbance values of the cells with and without the treatment of nanocomplexes, respectively. The [Abs]_blank_ are the absorbance of CCK-8 itself at 450 nm. All experiments were investigated in sextuplicate. Results were presented as mean ± standard deviation (SD).

### Visualization of CO_2_ Bubble Generation from NaGdF_4_@CaCO_3_-PEG nanoparticles

To observe the bubble generation characteristic of NaGdF_4_@CaCO_3_-PEG, the aqueous dispersion was dropped on a glass slide, followed by drying naturally. Then, PBS buffer with various pH values from 5.0 to 7.4 was dropped on the samples, and the CO_2_ bubble image from NaGdF_4_@CaCO_3_-PEG was obtained by an optical microscope at room temperature^10, 11^.

### *In Vitro* US Imaging at Various pH

*In vitro* US imaging of NaGdF_4_@CaCO_3_-PEG was performed in phosphate buffer solutions at various pH conditions (7.4, 7.0, 6.8, and 5.0)^41^. An optically transparent phantom gel plate, which was made by embedding a 500 μL Eppendorf tube in the agarose gel (3%, w/v) and then removing the tube after the phantom gel had cooled, was used for the *in vitro* experiments. Aqueous nanoparticle solutions (10 mg∙mL^−1^) were prepared at various pH. US images were obtained using Vevo 2100 imaging system operated at 21 MHz of a static state using a contrast mode. The change of US intensity for each sample was measured up to 180 min, and the US intensity of the water as control was subtracted from the sample intensity for the normalization.

### ***In Vitro*** MR Imaging at Various pH

*In vitro* MR imaging of NaGdF_4_@CaCO_3_-PEG was performed in phosphate buffer solutions at various pH conditions (7.4, 7.0, 6.8, and 5.0) using T_1_ -weighted MRI on a 0.5 T NMI20-Analyst NMR system (Niumag Corporation, Shanghai, China) to evaluate the contrast-enhancement effect^42^.

### Relaxivity and MRI phantom studies at 0.5 T magnetic field

A series of NaGdF_4_@CaCO_3_-PEG nanoparticle aqueous solutions with different Gd concentrations (12.5, 10, 7.5, 5.0, 2.5, and 1.25 mM) were prepared for MRI phantom and relaxivity studies. All experiments were performed on a 0.5 T NMI20-Analyst NMR system (Niumag Corporation, Shanghai, China)^42–44^. The longitudinal relaxation times (T_1_) were measured using an inversion recovery (IR) sequence. The longitudinal (r1) was determined from the slope of the plot of 1/T_1_ against the Gd concentration (mM).

### *In vivo* US imaging of Xenograft Tumor

To form a solid tumor in nude mice, the LN3 xenograft tumor was estabilished in 4-week-old male nude mice by injecting 10^6^ LN3 cells into the right thigh of mice. After injection, tumor-bearing nude mice were kept for 10~14 days to achieve a tumor size around 80 mm^3^. Then, 200 μL of normal saline (NS) containing NaGdF_4_@CaCO_3_-PEG (2 mg∙mL^−1^) was injected by an intratumoral injection. After injection, the tumor was imaged with the Vevo 2100 imaging system.

### ***In Vivo*** MR Imaging of Xenograft Tumor

For *in vivo* MRI measurements, LN3 tumor-bearing mice were intra-tumour injected with 200 μL of 2 mg∙mL^−1^ NaGdF_4_@CaCO_3_-PEG. At different intervals (0–3 h), T_1_-weighted MR images were observed using the rapid acquisition with relaxation enhancement sequence on 7.0 T small animal MRI scanner (Bruker Avance II 500WB spectrometer)^43^. Imaging parameters are as follows: repetition time, 2500 ms; echo time, 35 ms; rare factor, 8; field of view = 30 × 30mm^2^; image size, 256 × 256; slice thickness,0.7mm; and number of average, 2.

## Electronic supplementary material

Supplementary information

## References

[1] Jemal A (2011). Global cancer statistics. CA Cancer J Clin.

[2] Siegel RL (2016). Cancer statistics, 2016. CA Cancer J Clin.

[3] DeSantis CE (2016). Cancer statistics for African Americans, 2016: Progress and opportunities in reducing racial disparities. CA Cancer J Clin.

[4] Zhou Z (2015). Surface and Interfacial Engineering of Iron Oxide Nanoplates for Highly Efficient Magnetic Resonance Angiography. Acs Nano.

[5] Chen N (2014). Folic acid-conjugated MnO nanoparticles as a T_1_ contrast agent for magnetic resonance imaging of tiny brain gliomas. ACS Appl. Mater. Interfaces.

[6] Xing H (2014). Ultrasmall NaGdF_4_ Nanodots for Efficient MR Angiography and Atherosclerotic Plaque Imaging. Adv. Mater..

[7] Huang G (2014). Highly magnetic iron carbide nanoparticles as effective T_2_ contrast agents. Nanoscale.

[8] Huang J (2010). HSA coated MnO nanoparticles with prominent MRI contrast for tumor imaging. Chem. Commun..

[9] Liao N (2016). Poly (dopamine) coated superparamagnetic iron oxide nanocluster for noninvasive labeling, tracking, and targeted delivery of adipose tissue-derived stem cells. Sci. Rep.

[10] Kyung H (2015). pH-Controlled Gas-Generating Mineralized Nanoparticles: A Theranostic Agent for Ultrasound Imaging and Therapy of Cancers. Acs Nano.

[11] Kim M (2016). Nanosized Ultrasound Enhanced-Contrast Agent for *in Vivo* Tumor Imaging via Intravenous Injection. ACS Appl. Mater. Interfaces..

[12] Chang E (2015). F-18-FAZA PET Imaging Response Tracks the Reoxygenation of Tumors in Mice upon Treatment with the Mitochondrial Complex I Inhibitor BAY 87–2243. Clin Cancer Res.

[13] Jiang L (2014). A Radiofluorinated Divalent Cystine Knot Peptide for Tumor PET Imaging. Mol Pharmaceut.

[14] Persson M (2013). First F-18-labeled ligand for PET imaging of uPAR: *In vivo* studies in human prostate cancer xenografts. Nucl. Med. Biol..

[15] Nielsen CH (2010). PET Imaging of Tumor Neovascularization in a Transgenic Mouse Model with a Novel Cu-64-DOTA-Knottin Peptide. Cancer Res..

[16] Choi SH (2011). Large-Scale Synthesis of Bioinert Tantalum Oxide Nanoparticles for X-ray Computed Tomography Imaging and Bimodal Image-Guided Sentinel Lymph Node Mapping. J. Am. Chem. Soc..

[17] Shieh DB (2010). *In Vitro* and *in Vivo* Studies of FePt Nanoparticles for Dual Modal CT/MRI Molecular Imaging. J. Am. Chem. Soc..

[18] Rabin O (2006). An X-ray computed tomography imaging agent based on long-circulating bismuth sulphide nanoparticles. Nat. Mater..

[19] Wang L (2013). A Gd-doped Mg-Al-LDH/Au nanocomposite for CT/MR bimodal imagings and simultaneous drug delivery. Biomaterials.

[20] Lin LS (2014). Multifunctional Fe_3_O_4_@Polydopamine Core-Shell Nanocomposites for Intracellular mRNA Detection and Imaging-Guided Photothermal Therapy. Acs Nano.

[21] Huang Y (2016). Chitosan oligosaccharide based Gd-DTPA complex as a potential bimodal magnetic resonance imaging contrast agent. Magn. Reson. Imaging.

[22] Wang H (2016). Aerosol deposition in the lungs of spontaneously breathing rats using Gd‐DOTA‐based contrast agents and ultra‐short echo time MRI at 1.5 Tesla. Magn. Reson. Med..

[23] Randolph LM (2016). Polymeric Gd-DOTA amphiphiles form spherical and fibril-shaped nanoparticle MRI contrast agents. Chem. Sci.

[24] Chung MF (2012). A liposomal system capable of generating CO_2_ bubbles to induce transient cavitation, lysosomal rupturing, and cell necrosis. Angew Chem-Int Edit.

[25] Kang E (2010). Nanobubbles from Gas‐Generating Polymeric Nanoparticles: Ultrasound Imaging of Living Subjects. Angew Chem-Int Edit.

[26] Lee SJ (2011). Tumor-homing photosensitizer-conjugated glycol chitosan nanoparticles for synchronous photodynamic imaging and therapy based on cellular on/off system. Biomaterials.

[27] Zheng Z (2016). Using “On/Off” ^19^F NMR/Magnetic Resonance Imaging Signals to Sense Tyrosine Kinase/Phosphatase Activity *in Vitro* and in Cell Lysates. Anal. Chem..

[28] Miao Q (2016). Semiconducting Oligomer Nanoparticles as an Activatable Photoacoustic Probe with Amplified Brightness for *In Vivo* Imaging of pH. Adv. Mater..

[29] Yiguang W (2014). A nanoparticle-based strategy for the imaging of a broad range of tumours by nonlinear amplification of microenvironment signals. Nat. Mater..

[30] Mi P (2016). A pH-activatable nanoparticle with signal-amplification capabilities for non-invasive imaging of tumour malignancy. Nat. Nanotech.

[31] Liu Z (2011). Iron oxide nanoparticle-containing microbubble composites as contrast agents for MR and ultrasound dual-modality imaging. Biomaterials.

[32] Hou Y (2012). NaGdF_4_ nanoparticle-based molecular probes for magnetic resonance imaging of intraperitoneal tumor xenografts *in vivo*. Acs Nano.

[33] Johnson NJ (2011). Size-tunable, ultrasmall NaGdF_4_ nanoparticles: insights into their T_1_ MRI contrast enhancement. Chem. Mater..

[34] Wang F (2014). Preparation of core-shell NaGdF_4_ nanoparticles doped with luminescent lanthanide ions to be used as upconversion-based probes. Nat. Protoc..

[35] Dühnen S (2015). Size control of nearly monodisperse β-NaGdF_4_ particles prepared from small α-NaGdF_4_ nanocrystals. Chem. Mater..

[36] Jin X (2015). An ultrasmall and metabolizable PEGylated NaGdF_4_: Dy nanoprobe for high-performance T_1_/T_2_-weighted MR and CT multimodal imaging. Nanoscale.

[37] Brugiores P (1994). Randomised double blind trial of the safety and efficacy of two gadolinium complexes (Gd-DTPA and Gd-DOTA). Neuroradiology.

[38] Johnson NJJ (2011). Size-Tunable, Ultrasmall NaGdF_4_ Nanoparticles: Insights into Their T_1_ MRI Contrast Enhancement. Chem. Mater..

[39] Zhou C (2015). Aptamer CaCO_3_ Nanostructures: A Facile, pH-Responsive, Specific Platform for Targeted Anticancer Theranostics. Chem-Asian J.

[40] Liu X (2016). SPION@Cu_2−x_S nanoclusters for highly sensitive MRI and targeted photothermal therapy of hepatocellular carcinoma. J. Mater. Chem. B.

[41] Zeng Y (2014). Lipid-AuNPs@PDA nanohybrid for MRI/CT imaging and photothermal therapy of hepatocellular carcinoma. ACS Appl. Mater. Interfaces.

[42] Zhang D (2016). Lipid micelles packaged with semiconducting polymer dots as simultaneous MRI/photoacoustic imaging and photodynamic/photothermal dual-modal therapeutic agents for liver cancer. J. Mater. Chem. B.

[43] McDonagh BH (2016). L‐DOPA‐Coated Manganese Oxide Nanoparticles as Dual MRI Contrast Agents and Drug‐Delivery Vehicles. Small.

[44] Frangville C (2016). Hyperbranched polymer mediated size-controlled synthesis of gadolinium phosphate nanoparticles: colloidal properties and particle size-dependence on MRI relaxivity. Nanoscale.

