# Supplementary material for: Core-shell NaGdF4@CaCO3 nanoparticles for enhanced magnetic resonance/ultrasonic dual-modal imaging via tumor acidic micro-enviroment triggering
Source: Sci Rep. 2017 Jul 14;7:5370. doi: 10.1038/s41598-017-05395-w (PMC5511195; doi:10.1038/s41598-017-05395-w)
Supplement: Supplementary file 1 — Supplementary information [file 41598_2017_5395_MOESM1_ESM.pdf]

Supplementary information for "Core-shell $NaGdF_4@CaCO_3$ nanoparticles for enhanced magnetic resonance/ultrasonic dual-modal imaging via tumor acidic micro-enviroment triggering

Zuwu Wei [a,b#], *Xiao Lin* [c#], *Ming Wu* [a,b], *Bixing Zhao* [a,b], *Ruhui Lin* [d], *Da Zhang* [a,b], *Yun Zhang* [e], *Gang Liu* [f], *Xiaolong Liu* [a,b *], *Jingfeng Liu* [a,b,c*]

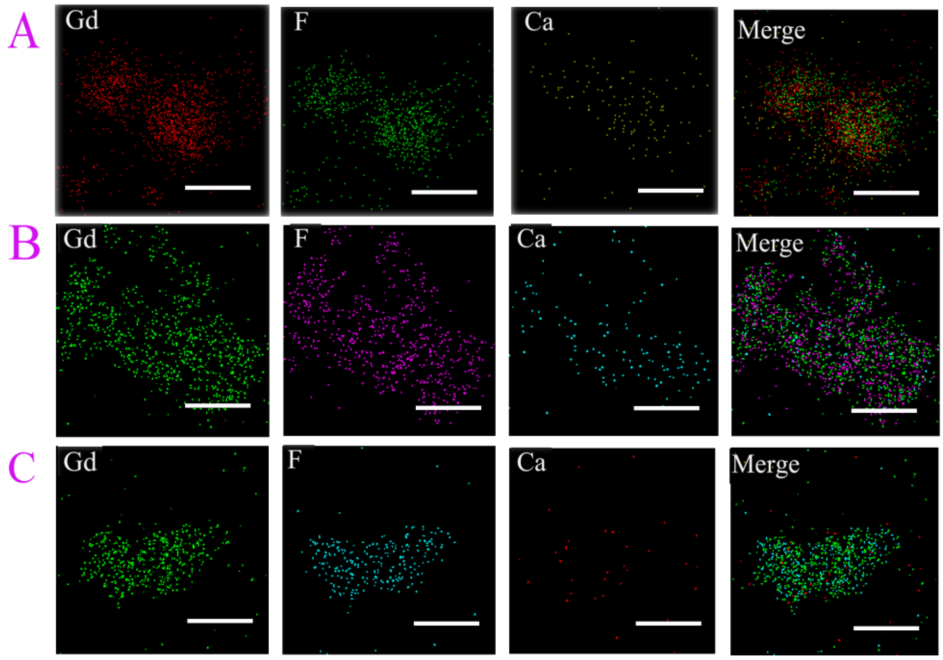

Figure S1 STEM image of $NaGdF_4@CaCO_3$-PEG (A), $NaGdF_4@CaCO_3$-PEG with a thicker shell (B) and core only $NaGdF_4$ and corresponding element mappings (for Gd, F and Ca), the bar is 100 nm.

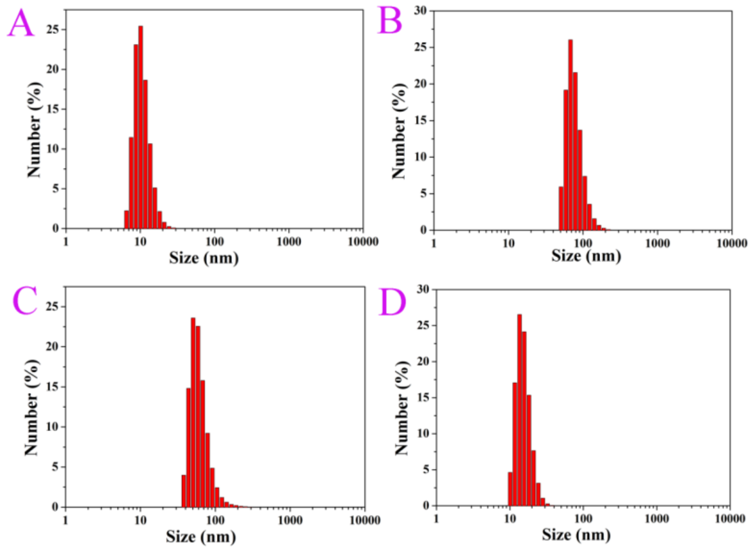

Figure S2 Size distributions of $NaGdF_4$ dispersed in cyclohexane (A); $NaGdF_4$ @$CaCO_3$ (B) and $NaGdF_4$ @$CaCO_3$-PEG (C) dispersed in H_2O; and $NaGdF_4$ @$CaCO_3$-PEG dispersed in PBS (pH 5.0) measured by DLS.

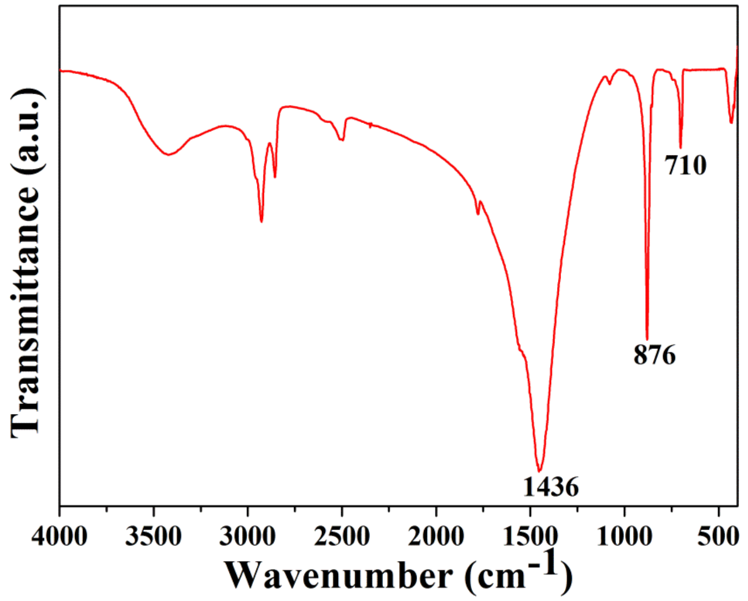

Figure S3 FT-IR spectra of $NaGdF_4$ @$CaCO_3$ with a thicker shell.

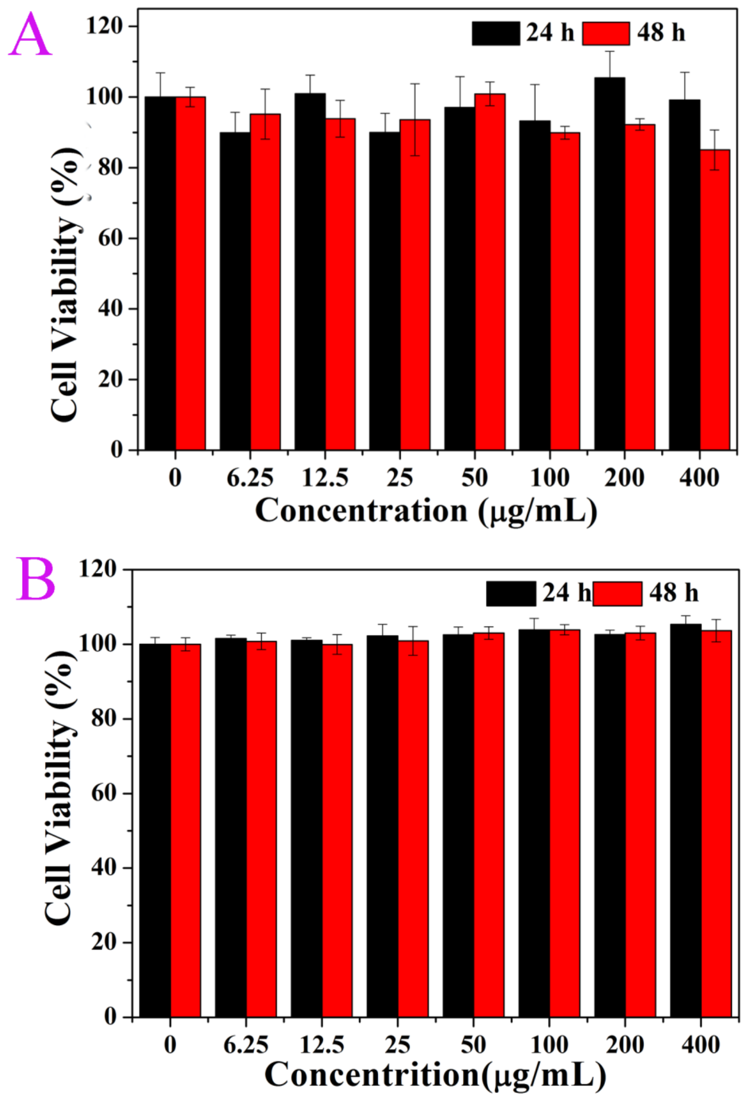

Figure S4 In vitro cell viability of LN3 (A) and NIH3T3 (B) cells incubated with $NaGdF_4@CaCO_3$-PEG at different concentrations for 24 h (Black) and 48 h (red).

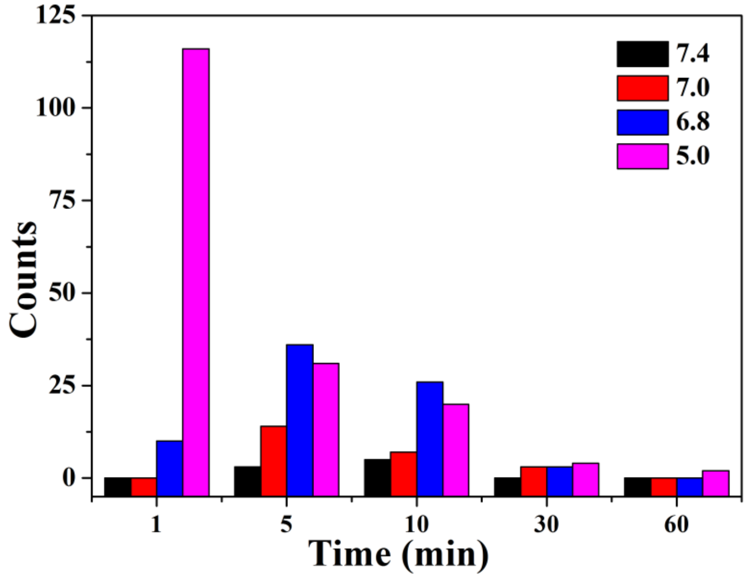

Figure S5 Quantification of generated bubbles in each image of Figure 4.

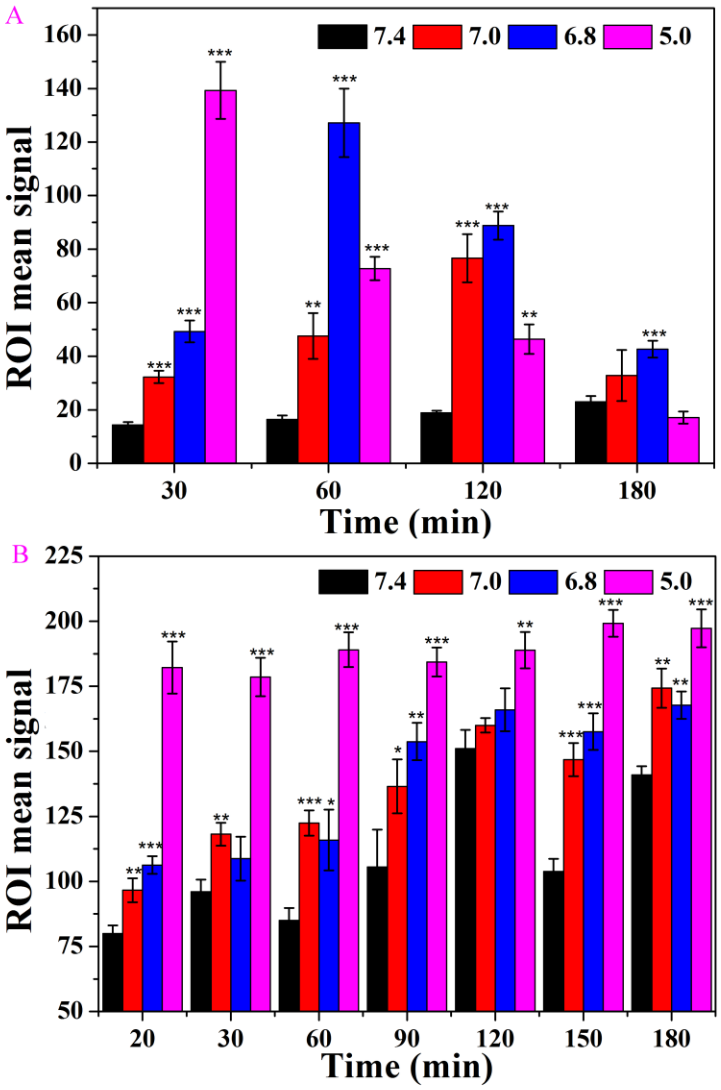

Figure S6 The gray values of Figure 5 (A) and 6 (B), p values were calculated using GraphPad Prism 6 (* $p < 0.05$, ** $p < 0.01$, *** $p < 0.001$; $n = 3$ per group).

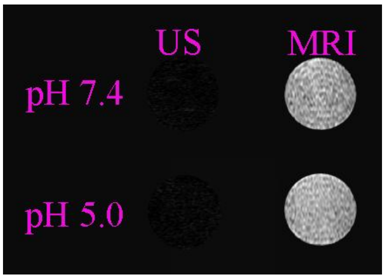

Figure S7 US and MRI images of $NaGdF_4$ at pH 6.8 and pH 7.4.
